# Biomass Thermogravimetric Analysis: Uncertainty Determination Methodology and Sampling Maps Generation

**DOI:** 10.3390/ijms11072701

**Published:** 2010-07-15

**Authors:** Jose A. Pazó, Enrique Granada, Ángeles Saavedra, Pablo Eguía, Joaquín Collazo

**Affiliations:** 1 ETS Ingenieros Industriales, University of Vigo, Lagoas-Marcosende s/n 36200–Vigo, Spain; E-Mails: jpazo@uvigo.es (J.A.P.); peguia@uvigo.es (P.E.); 2 ETS Ingeniería de Minas, University of Vigo. Lagoas-Marcosende s/n 36200–Vigo, Spain; E-Mail: saavedra@uvigo.es

**Keywords:** solid biofuel, sampling methodology, uncertainty, prompt analysis, TG analysis

## Abstract

The objective of this study was to develop a methodology for the determination of the maximum sampling error and confidence intervals of thermal properties obtained from thermogravimetric analysis (TG), including moisture, volatile matter, fixed carbon and ash content. The sampling procedure of the TG analysis was of particular interest and was conducted with care. The results of the present study were compared to those of a prompt analysis, and a correlation between the mean values and maximum sampling errors of the methods were not observed. In general, low and acceptable levels of uncertainty and error were obtained, demonstrating that the properties evaluated by TG analysis were representative of the overall fuel composition. The accurate determination of the thermal properties of biomass with precise confidence intervals is of particular interest in energetic biomass applications.

## 1. Introduction

According to the Kyoto Protocol [1] and the 2009 Copenhagen United Nations Climate Change Conference, climate change is a significant challenge, and actions must be taken to prevent any further increase in global temperature. Thus, renewable energy sources will play an increasingly important role in securing the European Union’s energy supply and providing sustainable development. Moreover, renewable energy sources also help to protect the environment. An increase in energy demand and atmospheric CO_2_, as well as the high cost and limited availability of fossil fuels, have led to the partial replacement of fossil fuels with biomass [2].

Knowledge of the chemical composition, thermal behavior and reactivity of biomass is essential for the effective design and operation of thermochemical conversion units. Thermoanalytical techniques, such as thermogravimetric analysis (TG) and derivative thermogravimetry (DTG), provide this information in a straightforward manner [2–4]. TG analyses are based on the volatilization rate of fuels, which is dependent on the heating rate applied to the sample and the type of fuel.

The intrinsic heterogeneity of biomass and the small amount of sample used in TG experiments makes it difficult to accurately determine the thermal properties of biomass; thus, to determine the characteristics of biomass with an acceptable, clearly defined level of uncertainty, a well-defined TG method must be developed. Many studies on the accuracy of TG experiments have been published [5–9], and various sampling methods have been proposed. Currently, TG methodologies are often based on small samples obtained from large batches; thus, careful reduction is necessary to prevent segregation and stratification problems [8]. In general, a good sampling method should be able to achieve a representative sample without being affected by the aforementioned problems.

A new methodology for the sampling of solid biomass and determination of error associated with the measurement of thermal properties was presented [10,11] and validated. This method is independent of the origin, appearance and packaging of the batch used to acquire samples. In the present study, the error associated with the aforementioned methodology as well as the confidence intervals of moisture, volatile matter, fixed carbon and ash content were determined. Moisture content affects the heating value of biomass, and ash determines the level of fouling and corrosion [12,13]. Moreover, volatile compounds influence the behaviour of the flame. The overall uncertainty of the measurements was defined, allowing us to determine the minimum number of samples necessary to achieve an acceptable level of reliability. Because the fixed carbon content can be calculated as a function of moisture, volatile matter and ash content, the uncertainties of these properties affect the uncertainty in the concentration of fixed carbon [11]. A comparative study on the mean values of the thermal properties and the corresponding uncertainties in TG experiments [11] was conducted, and a relationship was not observed. Moreover, the confidence level and error associated with the measurement of thermal properties were not well correlated.

## 2. Experimental

All materials were handled in the same laboratory by the same analyst. Because the materials were exposed to environmental conditions for less than half an hour, the effects of environmental variations in the properties of the materials were ignored (variations in temperature and relative humidity were considered insignificant over such a short period of time). Laboratory instruments were verified and calibrated to assure that the experimental methodology was accurate. Errors registered during the experiments were considered to be non-systematic errors and were related to the precision of the experiment. Thus, these errors were quantified in the total sampling error.

### 2.1. Materials

Several lignocellulosic materials derived from agricultural waste, energy crops and forestry materials were investigated; thus, the broad spectrum of solid biomass that can be used as fuel in combustion processes was evaluated. Agricultural materials (pine nut shells (Pns) and hazelnut shells (Hs)) were stored in large bags, and forestry (poplar pellets (Pp)) and agroenergetic crop (brassica pellets (Bp)) materials were stored in sacks.

### 2.2. Sampling and Reduction of the Samples

Depending on the material, sampled masses varied from 320 × 10^−3^ kg to 730 × 10^−3^ kg. Fuel samples were obtained from a tube sampler, which was designed according to the requirements specified in CEN/TS [14] and the work of Pierre Gy [15]. The sampling methodology used to obtain the fuel samples is described in the literature [10,11], along with the method used to reduce the samples. Table 1 shows the average weight of the samples selected for TG analysis. Tweezers were used to place the samples into the crucibles.

### 2.3. TG Analysis Methodology

All experiments were performed on a TG-DTA/DSC SETARAM Labsys electronic thermobalance, which can achieve a maximum temperature of 1600 °C and heating rates from 0.001 to 50 °C·min^−1^. To avoid heat and mass transfer limitations, approximately 20 × 10^−6^ kg of sample was used, and platinum crucibles without lids were employed. All experiments were initially conducted under an inert flow of nitrogen at a rate of 45 mL·min^−1^ to prevent the samples from oxidizing and to determine the concentration of moisture and volatile material. Subsequently, dry air (45 mL·min^−1^) was used to determine the ash content. The parameters of the thermal analysis are shown in Table 2.

Steps 1 to 4 were conducted to determine the moisture content, while steps 5 to 10 were performed to determine the concentration of volatile material. Lastly, steps 11 to 13 were conducted to determine the ash content of the biomaterials. Most of the steps were not directly related to the determination of moisture, volatile matter and ash content; rather, many steps were conducted to determine other thermal properties of the materials not discussed in the present paper.

The tested samples were weighed inside the crucible and uniformly distributed to avoid internal gradients of heat and gas concentration [3]. Alternatively, a temperature gradient inside the particles was not considered due to the small size and quantity of the samples [2,16]. Because the volatile content is strongly affected by the heating rate, the results were not compared to those from previous studies [10,11].

Moisture content was determined by heating the sample to 378 K in an N_2_ atmosphere until a constant weight was achieved. The moisture content (M) was obtained from the following equation: M = 100 × (m_1_ – m_2_)/m_1,_ where m_i_ (10^−6^ kg) is the difference between the initial mass (m_1_) of the sample and the constant mass (m_2_) at 378 K. The volatile matter was determined as the weight loss due to heating from 378 (step 5) to 873 K (step 10) in an N_2_ atmosphere. The volatile content (V) was calculated according to the following equation: V = 100 × (m_2_ – m_3_)/m_1_, where m_3_ (10^−6^ kg) is the mass of the sample at 873 K. Ash is the residual inorganic matter remaining after combustion, and the ash content was obtained from the equation A = 100 × m_4_/m_1_, where m_4_ (10^−6^ kg) is the mass remaining after step 13. Subsequently, the amount of fixed carbon (FC) was determined from the formula FC = 100 – M – V – A, where A, V and FC were calculated on a dry weight basis (db) and M was calculated on a wet basis (wb).

### 2.4. Statistical Treatment

#### 2.4.1. The Determination of the Maximum Error

The statistical treatment used in this study has been previously described [10,11,15]; thus, only a summary is presented in the current paper. Assuming that the sampling procedure is correct, the sampling error, *SE = (a_S_ - a_L_)/a_L_*, is a random variable with a mean of zero and a variance of *σ^2^(SE) = σ^2^(FE) + σ^2^(SGE)*; where *a_S_* is the value of a property from an individual sample, *a_L_* is the value of a property from the entire sample, *FE* is the fundamental error and *SGE* is the segregation and grouping error. A proper sampling technique leads to an accurate experimental procedure; thus, the sampling error is related to the precision of the experiment. For simplicity, the sample element is assumed to be a dimensionless unit of mass in the equations shown below; however, this can only be assumed when the sample elements possess a similar mass. The sample mass is represented as n, the number of elements in the sample, while N_F_ is the mass of the entire sample. The variance in the fundamental error can be expressed as:

(1) $$\sigma^{2}(FE)=\left(\frac{1}{n}-\frac{1}{N_{F}}\right)\cdot {HI}_{L}\approx \frac{1}{n}{HI}_{L}$$

where *HI_L_* is the heterogeneity invariant, and the variance of the sampling error is

(2) $$\sigma^{2}(SE)\leq 2\sigma^{2}(FE)=2\left(\frac{1}{n}-\frac{1}{N_{F}}\right)\cdot {HI}_{L}\approx \frac{2}{n}{HI}_{L}$$

Assuming that the sampling error follows a normal distribution (*SE ∼ N(0,σ(SE))*, as Central Limit Theorem states, we can ensure with a confidence level of 95% that

(3) $$|SE|\leq {SE}_{\text{max}}=1.96\sqrt{\frac{2{HI}_{L}}{n}}$$

and

(4) $$n_{\text{min}}\geq 7.68\frac{{HI}_{L}}{{SE}_{\text{max}}^{2}}$$

where *SE_max_* is the upper bound of the sampling error for a given sampling size (n), and n_min_ is the minimum sampling size for a given sampling error. Because moisture, volatile matter and ash content are measured variables, *SE_max_* represents the maximum sampling error. The amount of fixed carbon (*FC*) was obtained directly from the properties of the materials, $\bar{FC}\;=\;\left(100-\bar{\text{M}}-\bar{\text{V}}-\bar{\text{A}}\right)$, and the maximum error was calculated by the method of error propagation, which is fully described in the literature [11]:

(5) $${SE}_{\text{max}}(FC)=\sqrt{\frac{7.68}{M_{m}}\times \frac{{\bar{M}}^{2}\;{HI}_{L}(M)+{\bar{V}}^{2}\;{HI}_{L}(V)+{\bar{A}}^{2}\;{HI}_{L}(A)}{{\left(100-\bar{M}-\bar{V}-\bar{A}\right)}^{2}}}$$

*M̅*, *V̅*, *A̅* and $\bar{FC}$ are the average moisture, volatile matter, ash and fixed carbon content, respectively.

#### 2.4.2. Determination of Confidence Intervals

Another objective of this study was to approximate *a_L_*, the value of a property in the entire sample. Assuming that *a_S_* follows a normal distribution and that the sampling procedure is correct, *a_S_* is a random variable with the following distribution:

(6) $$a_{S}∼N(a_{L},\sigma (a_{s}))$$

From the definition of the sampling error and equation 2, an approximation of the variance of *a_S_* was obtained:

(7) $$\sigma^{2}(a_{S})=\sigma^{2}(SE)\times a_{L}^{2}\leq \frac{2}{n}{HI}_{L}\times a_{L}^{2}\approx \frac{2}{n}{HI}_{L}\times a_{S}^{2}$$

Finally, the value of a property in the entire sample, *a_L_*, can be estimated from the mean experimental values, and confidence intervals for *a_L_* can be obtained from the approximation of the variance and the mean of *a_S_*.

## 3. Results and Discussion

Moisture (*wb*), volatile matter (*db*), fixed carbon (*db*) and ash content (*db*) of the samples are presented in Table 3, including the mean and variance of each variable. As shown in the table, brassica displayed a high ash content.

*HI_L_*, the heterogeneity invariant, was calculated according to the method described in Section 2.4.1. and is summarized in Table 4. The maximum sampling error of a sample with a fixed mass was obtained from the *HI_L_*, and the results indicated that the minimum sample size corresponded to a fixed sampling error. The minimum sample size and maximum sampling error associated with the determination of moisture, volatile matter, fixed carbon and ash content are provided in Tables 5 and 6, 7 and 8, 9 and 10, and 11 and 12, respectively.

To show the utility of the results displayed in Tables 5, 7, 9 and 11, the minimum sample mass required to achieve an accurate representation of the moisture content of hazelnut shells (Hs) was determined. A maximum sampling error of 0.05 was selected, and the corresponding non-dimensional sample size was 14.70, as shown in Table 5. The minimum sampling size was subsequently multiplied by the average weight of Hs samples (21.29 × 10^−6^ kg) to provide a minimum sample weight of 312.9 × 10^−6^ kg.

To demonstrate the use of Tables 6, 8, 10 and 12, an inverse calculation of the previous example was performed. The maximum sampling error of a sample with a mass of 312.9 × 10^−6^ kg was determined by dividing the sample mass by the average weight of Hs samples (21.29 × 10^−6^ kg), and a value of 14.7 was obtained. The maximum sampling error was calculated from the equation ${SE}_{\text{max}}=6.07\times 10^{-2}\sqrt{10/14.7}=0.05$. Using the methodology described in section 2.4.1, Tables 5–12 were generated with a confidence level of 95%.

According to the methodology described in Section 2.4.2, confidence intervals for the properties of each material were generated. As an example, the determination of the confidence intervals of the moisture content of hazelnut shells (Hs) is illustrated. According to the data shown in Table 3, the mean moisture content of Hs is 10.873. Moreover, the results displayed in Table 4 indicate that the *HI_L_* of Hs is 4.79 × 10^−3^. In this example, nine samples were tested; thus, *σ^2^(a_S_)* ≈ *(2/n)*·*HI_L_*·*a_S_^2^* = 0.126. According to the methodology described in Section 2.4.2., the confidence intervals of the moisture content of Hs are $10.87\pm 1.96\sqrt{0.126}=10.87\pm 0.695$. The confidence intervals of all of the materials and associated properties were calculated at a 95% confidence level, as shown in Table 13. To compare the results of the present to those of previous studies, confidence intervals for the prompt analysis presented in the literature [11] were calculated. The mean weights of the samples in TG analysis were approximately 1000-times less than those of the prompt analysis [11]; thus, the confidence intervals of TG should be significantly wider ( $\sqrt{1000}=31.623$). However, the accuracy of TG equipment compensates for a smaller sample weight, leading to confidence intervals that are approximately five-times greater than those of the prompt analysis.

**Figure 1.**
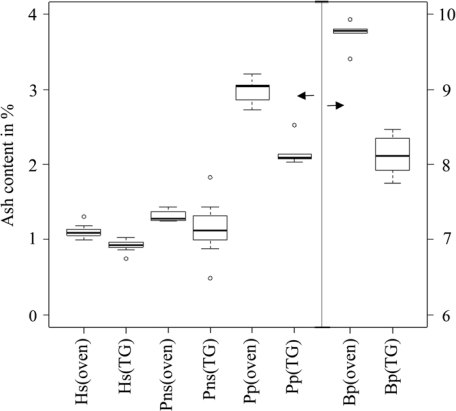
Box-plots of the TG (TG) and prompt (oven) analysis [11] of ash content. Left y axis scale for Hs, Pns and Pp, and right y axis scale for Bp. Symbol “O” represents outliers.

Volatile matter and fixed carbon contents obtained from the TG and prompt analysis are not comparable because the results are dependent on the thermal history of the particles, which are completely different in the prompt and TG analysis. However, the moisture content of the materials should be comparable. Lignocellulosic biomass is mainly composed of cellulose, hemicellulose and lignin. At low heating rates, cellulose begins to decompose at temperatures greater than 300 ºC [17], and hemicellulose begins to decompose at 220 ºC. However, lignin decomposes very slowly over a wide temperature range, beginning at 160 ºC [18]. Because the moisture content was determined at temperatures below 378 K (Table 2), it was assumed that water was not produced through pyrolysis; thus, the results of the present study should be comparable to those of the prompt analysis. As shown in Table 13, the mean moisture content obtained in the TG analysis was lower than the mean moisture content of the prompt analysis. Moreover, the mean ash content obtained from TG analysis was lower than the mean ash content of the prompt analysis. A box-plot of ash content illustrating the median, outliers, smallest and largest observation, and lower and upper quartiles are shown in Figure 1. The results indicated that the ash content obtained from the TG and prompt analyses were not comparable due to the methodology of the TG analysis. The ash content obtained from TG analysis was uniformly lower than that of the prompt analysis; thus, biomass heterogeneity was an unlikely cause for the discrepancy in the results. Due to the low sample weight (20 × 10^−6^ kg), TG crucibles were loaded with tweezers. It is possible that big particles were favored in this process, and small particles and dust were effectively removed.

To verify the aforementioned hypothesis, the TG analysis was conducted on biomass with a small particle size (<0.3 × 10^−3^ m). As shown in Table 14, the ash content of fine particles was greater than the ash content of the original materials in the TG and prompt analyses. It is not possible to assure that the particle size distribution of the materials in the TG analysis is identical to that of the prompt analysis; therefore, the mean ash content of these methods is not comparable. A similar explanation is proposed for the determination of moisture content; however, handling particles smaller than 0.3 × 10^−3^ m may affect the results. Thus, it was not feasible to validate this hypothesis. In general, these results indicate that the mean ash and moisture content obtained from the TG and prompt analysis are not comparable when the proposed methodology is applied.

Correlations between the moisture, volatile matter and ash content of the materials (fixed carbon was calculated from these properties) were observed at a confidence level of *α* = 0.05. The ash and moisture content of Hs displayed a Pearson correlation coefficient of 0.69, and the moisture and volatile content of Pp displayed a correlation coefficient of 0.89. Thus, for all other properties and materials, the value of one variable cannot be explained by other variables because the properties are not linearly related. All three variables must be studied separately, and the analysis of one property cannot be used to infer the value of others. Based on the results of the prompt analysis, a similar conclusion was made [11].

Although the properties of TG and prompt analysis are not related, a relationship between the maximum sampling error can be extrapolated from one analysis to the other. The maximum sampling error of the materials from the prompt analysis [11] was extrapolated to the TG analysis, and the extrapolated error was greater than the maximum sampling error obtained from TG analysis. To illustrate this result, the error associated with the moisture content of hazelnut shells (Hs) was extrapolated. According to the literature results [11], *HI_L_* = 9.21 × 10^−5^ and the maximum sampling error for a sample with an average weight of 21.7 × 10^−3^ kg is 2.66 × 10^−2^. By taking into account the relationship between the average weights of both analyses, the maximum sampling error of TG analysis was estimated:

(8) $${\hat{SE}}_{\text{max}}(TG)=\sqrt{7.68\times 9.21\times 10^{-5}\times \frac{21.7\times 10^{-3}\text{kg}}{21.29\times 10^{-6}\text{kg}}=0.721}$$

**Figure 2.**
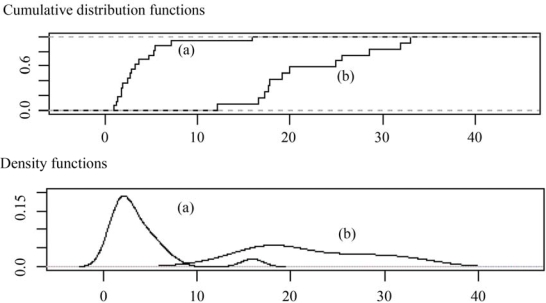
(**a**) Empirical distribution of ***SE_max_(TG)/SE_max_(prompt)*** (**b**) density functions of ${\hat{\text{SE}}}_{\text{max}}(\text{TG})/{\text{SE}}_{\text{max}}(\text{prompt})$.

This result does not agree with those shown in Table 6 of the present paper, where *SE_max_(TG)* = 1.92 × 10^−1^. The analysis was repeated for all of the materials and properties, and values of *SE_max_(TG)/SE_max_(prompt)* varied vary from 1 to 16, while values of ${\hat{SE}}_{\text{max}}(TG)/{SE}_{\text{max}}(\text{prompt})$ varied from 12 to 33. The empirical distribution and density functions of both quotients are shown in Figure 2, and the results suggested that *SE_max_(TG)* cannot be estimated from *SE_max_(prompt). SE_max_(TG)/SE_max_(prompt)* reached a maximum value of 16 because atypical values were present in the density distribution function (Figure 2 (a)). However, when atypical values were removed, the maximum quotient was equal to 7. The *HI_L_* of the TG and prompt analysis are very different, which corroborates the lack of a relationship between the maximum sampling errors of the methods. As shown previously, the maximum sampling error of the TG analysis should be significantly greater (12–33 times) than that of the prompt analysis, but the accuracy of TG equipment compensates for the small sample weight, leading to maximum sampling errors that are approximately 1–7 times greater than *SE_max_(prompt).*

To observe other relationships between *SE_max_(TG)* and *SE_max_(prompt),* a general correlation study was conducted on the sampling error associated with the volatile matter, fixed carbon and ash content and the corresponding *SE_max_(prompt)* [11]. A significant correlation was observed, and a correlation coefficient of 0.63 and a p-value of 0.028 were obtained. Although the correlation is significant, the low value of the correlation coefficient suggests high levels of error will be encountered if *SE_max_(TG)* is estimated from *SE_max_(prompt)*.

## 4. Conclusions

This study provided a statistical analysis of the sampling error or level of uncertainty associated with the properties measured in a TG analysis as well as the corresponding confidence intervals. This information can be used in any granular material processing application where accuracy is important. Moreover, statistical analysis is crucial for determining the propagation of error in future calculations. The sampling procedure and statistical techniques used in this study can be extrapolated to any other solid material in granular form that possesses a homogeneous particle size distribution. Although biofuels are heterogeneous materials, the materials evaluated in this investigation showed reasonable limits. Despite the heterogeneity of biofuels, a well-planned selection of samples can lead to an extrapolation of sample properties from a large batch, and a controlled, analyzed, quantified level of uncertainty can be achieved.

Although the mean weights of the samples in TG analysis were small, the accuracy of TG equipment compensated for a low sample weight, leading to confidence intervals that were smaller than expected.

The results of TG analysis were compared to those of a prompt analysis, and the results suggested that the mean values and maximum sampling errors were not correlated. Thus, the mean and error obtained from one analysis cannot be used to estimate the mean or error associated with another analysis.

## Figures and Tables

**Table 1.** Average weights of samples selected for TG analysis.

**Material**	**Sample Weight (kg)**
Hazelnut shell (Hs)	21.29 × 10^−6^
Pine nut shell (Pns)	21.98 × 10^−6^
Poplar pellets (Pp)	22.46 × 10^−6^
Brassica pellets (Bp)	21.18 × 10^−6^

**Table 2.** Thermal evolution of the samples in the TG experiments.

**Step**	**1**	**2**	**3**	**4**	**5**	**6**	**7**	**8**	**9**	**10**	**11**	**12**	**13**
**T_start_ (K)**	303	343	363	378	378	418	418	773	773	873	873	873	973
**T_end_ (K)**	343	363	378	378	418	418	773	773	873	873	873	973	973
SR* (K/min)	30	15	2	0	10	0	10	0	20	0	0	20	0
**Time (s)**	80	80	450	1800	240	600	2130	3600	300	600	2400	300	600
**Atmosphere**	N_2_	N_2_	N_2_	N_2_	N_2_	N_2_	N_2_	N_2_	N_2_	N_2_	Air	Air	Air

* Scan Rate.

**Table 3.** The moisture, volatile matter, fixed carbon and ash content of each type of biomass. Except for moisture content, all values are reported on a dry weight basis.

**Samples 1 to 5:**						
**Material**	**Property**	**Sample 1**	**Sample 2**	**Sample 3**	**Sample 4**	**Sample 5**
**Hs**	Moisture	10.015	9.498	11.495	11.775	11.055
	Volatiles	67.421	69.388	68.587	69.938	70.688
	Fixed Carbon	31.613	29.861	30.519	29.102	28.417
	Ash	0.966	0.751	0.894	0.960	0.895
**Pns**	Moisture	10.952	11.528	12.367	11.570	12.758
	Volatiles	66.898	68.481	67.694	68.361	66.100
	Fixed Carbon	31.274	30.077	30.991	30.756	33.413
	Ash	1.828	1.442	1.316	0.883	0.487
**Pp**	Moisture	6.414	6.871	7.231	7.338	7.310
	Volatiles	75.227	75.816	76.369	77.307	77.812
	Fixed Carbon	22.679	22.144	21.107	20.563	20.111
	Ash	2.094	2.040	2.525	2.130	2.077
**Bp**	Moisture	8.819	8.309	8.679	9.890	9.288
	Volatiles	71.243	68.253	69.536	68.360	69.318
	Fixed Carbon	21.005	23.819	22.003	23.287	22.565
	Ash	7.752	7.928	8.461	8.353	8.116

**Samples 6 to 9, mean and variance:**							
**Material**	**Property**	**Sample 6**	**Sample 7**	**Sample 8**	**Sample 9**	**Mean**	**S^2^**
**Hs**	Moisture	11.335	10.152	11.577	10.951	10.873	0.637
	Volatiles	68.497	69.289	68.888	69.116	69.090	0.854
	Fixed Carbon	30.509	29.849	30.087	29.956	29.990	0.812
	Ash	0.995	0.861	1.025	0.928	0.919	0.007
**Pns**	Moisture	11.657	10.969	10.490	11.422	11.524	0.496
	Volatiles	67.392	67.961	67.712	66.758	67.484	0.610
	Fixed Carbon	31.495	30.865	31.296	32.116	31.365	0.901
	Ash	1.112	1.174	0.992	1.126	1.151	0.139
**Pp**	Moisture					7.033	0.155
	Volatiles					76.506	1.119
	Fixed Carbon					21.321	1.151
	Ash					2.173	0.040
**Bp**	Moisture					8.997	0.372
	Volatiles					69.342	1.451
	Fixed Carbon					22.536	1.210
	Ash					8.122	0.086

**Table 4.** The intrinsic heterogeneity of the moisture, volatile matter, fixed carbon and ash content of different biomass materials.

	***HI_L_***			
	**Moisture**	**Volatiles**	**Fixed Carbon**	**Ash**
**Hs**	4.79 × 10^−3^	1.59 × 10^−4^	8.02 × 10^−4^	7.06 × 10^−3^
**Pns**	3.32 × 10^−3^	1.19 × 10^−4^	8.14 × 10^−4^	9.31 × 10^−3^
**Pp**	2.50 × 10^−3^	1.53 × 10^−4^	2.03 × 10^−3^	6.71 × 10^−3^
**Bp**	3.68 × 10^−3^	2.41 × 10^−4^	1.91 × 10^−3^	1.04 × 10^−3^

**Table 5.** The minimum sample mass (expressed as n_min_ sampling units) required to achieve a predetermined maximum sampling error for the determination of moisture content.

		**Minimum sample size for a determined sampling error**			
		**Hs**	**Pns**	**Pp**	**Bp**
	***HI_L_***	4.79 × 10^−3^	3.32 × 10^−3^	2.50 × 10^−3^	3.68 × 10^−3^
**Maximum error**	0.001	3.68 × 10^4^	2.55 × 10^4^	1.92 × 10^4^	2.83 × 10^4^
	0.005	1.47 × 10^3^	1.02 × 10^3^	7.69 × 10^2^	1.13 × 10^3^
	0.01	3.68 × 10^2^	2.55 × 10^2^	1.92 × 10^2^	2.83 × 10^2^
	0.05	14.70	10.20	7.69	11.30

**Table 6.** The maximum sampling error, *SE_max_,* that corresponds to a given sample mass (expressed as n sampling units) for the determination of moisture content.

		**Maximum error for the sample size**			
		**Hs**	**Pns**	**Pp**	**Bp**
	***HI_L_***	4.79 × 10^−3^	3.32 × 10^−3^	2.50 × 10^−3^	3.68 × 10^−3^
**Sample size**	1	1.92 × 10^−1^	1.60 × 10^−1^	1.39 × 10^−1^	1.68 × 10^−1^
	10	6.07 × 10^−2^	5.05 × 10^−2^	4.38 × 10^−2^	5.32 × 10^−2^
	100	1.92 × 10^−2^	1.60 × 10^−2^	1.39 × 10^−2^	1.68 × 10^−2^
	200	1.36 × 10^−2^	1.13 × 10^−2^	9.80 × 10^−2^	1.19 × 10^−2^

**Table 7.** The minimum sample mass (expressed as n_min_ sampling units) that corresponds to a predetermined maximum sampling error for the determination of volatile matter content.

		**Minimum sample size for a determined sampling error**			
		**Hs**	**Pns**	**Pp**	**Bp**
	***HI_L_***	1.59 × 10^−4^	1.19 × 10^−4^	1.53 × 10^−4^	2.41 × 10^−4^
**Maximum error**	0.001	1.22 × 10^3^	9.15 × 10^2^	1.18 × 10^3^	1.85 × 10^3^
	0.005	48.90	36.60	47.00	74.20
	0.01	12.20	9.15	11.80	18.50
	0.05	4.89 × 10^−1^	3.66 × 10^−1^	4.70 × 10^−1^	7.42 × 10^−1^

**Table 8.** The maximum sampling error, *SE_max_*, that corresponds to a given sample mass (expressed as n sampling units) for the determination of volatile matter content.

		**Maximum error for the sample size**			
		**Hs**	**Pns**	Pp	**Bp**
	***HI_L_***	1.59 × 10^−4^	1.19 × 10^−4^	1.53 × 10^−4^	2.41 × 10^−4^
**Sample size**	1	3.50 × 10^−2^	3.03 × 10^−2^	3.43 × 10^−2^	4.31 × 10^−2^
	10	1.11 × 10^−2^	9.57 × 10^−3^	1.08 × 10^−2^	1.36 × 10^−2^
	100	3.50 × 10^−3^	3.03 × 10^−3^	3.43 × 10^−3^	4.31 × 10^−3^
	200	2.47 × 10^−3^	2.14 × 10^−3^	2.42 × 10^−3^	3.04 × 10^−3^

**Table 9.** The minimum sample mass required for the determination of fixed carbon content (expressed as n_min_ sampling units) for a predetermined maximum sampling error.

		**Minimum sample size for a determined sampling error**			
		**Hs**	**Pns**	**Pp**	**Bp**
	***HI_L_***	8.02 × 10^−4^	8.14 × 10^−4^	2.03 × 10^−3^	1.91 × 10^−3^
**Maximum error**	0.001	6.16 × 10^3^	6.26 × 10^3^	1.56 × 10^4^	1.46 × 10^4^
	0.005	2.47 × 10^2^	2.50 × 10^2^	6.23 × 10^2^	5.86 × 10^2^
	0.01	61.60	62.60	1.56 × 10^2^	1.46 × 10^2^
	0.05	2.47	2.50	6.23	5.86

**Table 10.** The maximum sampling error, *SE_max_*, that corresponds to a given sample mass (expressed as n sampling units) for the determination of fixed carbon content.

		**Maximum error for the sample size**			
		**Hs**	**Pns**	**Pp**	**Bp**
	***HI_L_***	8.02 × 10^−4^	8.14 × 10^−4^	2.03 × 10^−3^	1.91 × 10^−3^
**Sample size**	1	7.85 × 10^−2^	7.91 × 10^−2^	1.25 × 10^−1^	1.21 × 10^−1^
	10	2.48 × 10^−2^	2.50 × 10^−2^	3.94 × 10^−2^	3.83 × 10^−2^
	100	7.85 × 10^−3^	7.91 × 10^−3^	1.25 × 10^−2^	1.21 × 10^−2^
	200	5.55 × 10^−3^	5.59 × 10^−3^	8.82 × 10^−3^	8.56 × 10^−3^

**Table 11.** The minimum sample mass required for the determination of ash content (expressed as n_min_ sampling units) for a predetermined maximum sampling error.

		**Minimum sample size for a determined sampling error**			
		**Hs**	**Pns**	**Pp**	**Bp**
	***HI_L_***	7.06 × 10^−3^	9.31 × 10^−2^	6.71 × 10^−3^	1.04 × 10^−3^
**Maximum error**	0.001	5.42 × 10^4^	7.15 × 10^5^	5.16 × 10^4^	7.99 × 10^3^
	0.005	2.17 × 10^3^	2.86 × 10^4^	2.06 × 10^3^	3.20 × 10^2^
	0.01	5.42 × 10^2^	7.15 × 10^3^	5.16 × 10^2^	79.90
	0.05	21.70	2.86 × 10^2^	20.60	3.20

**Table 12.** The maximum sampling error, *SE_max_*, that corresponds to a given sample mass (expressed as n sampling units) for the determination of ash content.

		**Maximum error for the sample size**			
		**Hs**	**Pns**	**Pp**	**Bp**
	***HI_L_***	7.06 × 10^−3^	9.31 × 10^−2^	6.71 × 10^−3^	1.04 × 10^−3^
**Sample size**	1	2.33 × 10^−1^	8.46 × 10^−1^	2.27 × 10^−1^	8.94 × 10^−2^
	10	7.37 × 10^−2^	2.67 × 10^−1^	7.18 × 10^−2^	2.83 × 10^−2^
	100	2.33 × 10^−2^	8.46 × 10^−2^	2.27 × 10^−2^	8.94 × 10^−3^
	200	1.65 × 10^−2^	5.98 × 10^−2^	1.61 × 10^−2^	6.32 × 10^−3^

**Table 13.** Confidence intervals for the TG and prompt analysis of Moisture (wb), volatile matter (db), fixed carbon (db) and ash (db) content [11].

		**Moisture**	**Volatiles**	**Fixed Carbon**	**Ash**
**Hs**	TG	10.87 ± 6.95 × 10^−1^	69.09 ± 8.05 × 10^−1^	29.99 ± 7.85 × 10^−1^	0.92 ± 7.14 × 10^−2^
	Prompt	12.04 ± 1.07 × 10^−1^	73.57 ± 1.64 × 10^−1^	22.27 ± 1.22 × 10^−1^	1.11 ± 8.23·10^−2^
**Pns**	TG	11.52 ± 6.14 × 10^−1^	67.48 ± 6.80 × 10^−1^	31.36 ± 8.27 × 10^−1^	1.15 ± 3.25 × 10^−1^
	Prompt	12.63 ± 2.36 × 10^−1^	76.16 ± 3.08 × 10^−1^	19.73 ± 2.97 × 10^−1^	1.32 ± 6.89 × 10^−2^
**Pp**	TG	7.03 ± 4.36 × 10^−1^	76.51 ± 1.17	21.32 ± 1.19	2.17 ± 2.21 × 10^−1^
	Prompt	7.92 ± 1.71 × 10^−1^	80.43 ± 6.51 × 10^−1^	15.28 ± 6.77 × 10^−1^	2.98 ± 2.07 × 10^−1^
**Bp**	TG	9.00 ± 6.77 × 10^−1^	69.34 ± 1.34	22.54 ± 1.22	8.12 ± 3.25 × 10^−1^
	Prompt	10.13 ± 2.10 × 10^−1^	74.21 ± 8.99 × 10^−2^	14.44 ± 2.40 × 10^−1^	9.73 ± 2.17 × 10^−1^

**Table 14.** The mean ash content (% db) of the original samples and fine particles (<0.3 × 10^−3^ m) obtained from prompt and TG analyses.

	**Hs**	**Pns**	**Pp**	**Bp**
**Ash TG**	0.92	1.15	2.17	8.12
**Ash prompt**	1.11	1.32	2.98	9.73
**Ash TG (dust)**	2.98	3.37	3.91	10.94
